# Comparison of Refractive Outcomes After Phacoemulsification and Combined 25-Gauge Phacovitrectomy with Implantation of Plate-Haptic Toric Intraocular Lenses

**DOI:** 10.3390/jcm13226861

**Published:** 2024-11-14

**Authors:** Lara Buhl, Julian Langer, Franziska Kruse, Niklas Mohr, Thomas Kreutzer, Wolfgang Mayer, Stefan Kassumeh, Siegfried Priglinger

**Affiliations:** Department of Ophthalmology, University Hospital, LMU Munich, 80336 Munich, Germany; lara.buhl@med.uni-muenchen.de (L.B.); julian.langer@med.uni-muenchen.de (J.L.); franziska.kruse@med.uni-muenchen.de (F.K.); niklas.mohr@med.uni-muenchen.de (N.M.); thomas.kreutzer@med.uni-muenchen.de (T.K.); wolfgang.mayer@med.uni-muenchen.de (W.M.); stefan.kassumeh@med.uni-muenchen.de (S.K.)

**Keywords:** phacovitrectomy, toric intraocular lens, intraocular lens stability, cataract surgery, plate-haptic

## Abstract

**Objectives**: To compare intraocular lens (IOL) position and refractive outcomes between eyes that underwent sole phacoemulsification with those that underwent combined 25-gauge phacovitrectomy with a plate-haptic toric IOL implantation. **Methods**: This retrospective study included 60 eyes of 60 patients. Of these, 30 eyes underwent 25-gauge phacovitrectomy, while the other 30 eyes received phacoemulsification alone. In both groups, a plate-haptic toric intraocular lens (AT Torbi 709M, Carl Zeiss Meditec AG) was implanted. The main outcome measures were the refractive outcome, the refraction prediction error (PE), the difference in the postoperative anatomical lens position (ALP) change, and rotational stability. **Results**: The mean spherical equivalent decreased considerably from −2 ± 4.4 diopters (D) to −0.6 ± 1.4 D after phacovitrectomy (*p* = 0.05) and −0.7 D ± 5.5 D to −0.1 ± 1.1 D after phacoemulsification (*p* = 0.5). The prediction error (PE) was comparable between the two groups for all formulas (Haigis-T: *p* = 0.8, Barrett TK Toric: *p* = 0.8, Z CALC: *p* = 0.7). No significant difference in absolute ALP change and postoperative rotational stability was observed between the phacovitrectomy and phacoemulsification group (1.4 mm vs. 1.4 mm, *p* = 0.96; 2.9° vs. 2.1°, *p* = 0.5). **Conclusions**: The implantation of plate-haptic toric IOLs in the combined phacovitrectomy group resulted in refraction and IOL positioning outcomes comparable to those in the phacoemulsification-only group.

## 1. Introduction

Simultaneous phacovitrectomy is regularly conducted in patients with vitreoretinal pathologies and cataracts, providing rapid postoperative visual recovery and no need for additional lens surgery [1]. The procedure is widely regarded as safe and effective, yet there is increasing demand for the best possible postoperative refractive result [2]. Thus, in patients with preoperative astigmatism higher than one diopter (D), the implantation of a toric intraocular lens (IOL) should be considered before phacovitrectomy. However, this may also add to the complexity of refractive result precision, as the axial and rotational IOL stability can be a concern.

IOL power calculation in patients receiving combined phacovitrectomy can be challenging. The refractive outcome is considered less predictable, often resulting in postoperative myopic overcorrection [3,4,5,6]. The myopic shift is often attributed to the forward displacement of the IOL due to intraocular gas endotamponade or inaccurate axial length measurements using ultrasound [7,8]. In contrast, gas endotamponade can also lead to a more posterior IOL position due to enhanced zonular elasticity once the gas is absorbed [9]. Other factors related to IOL displacement after phacovitrectomy may include additional surgical stress on the capsular bag and enhanced postoperative capsular contraction due to increased inflammation following combined surgery [10,11].

Multiple factors impact not only the axial IOL position but also the postoperative rotational stability including the axial length, capsular bag size, capsulorhexis size and IOL properties [12]. Previous studies evaluated the rotational stability of loop-haptic toric IOL after combined phacovitrectomy, proving to be comparable to sole phacoemulsification [13,14]. We recently showed similar results utilizing a plate-haptic IOL with hydrophobic surface properties following phacovitrectomy [15]. However, comparability with the existing literature values is certainly limited, as differences in procedures, surgical subtleties and postoperative management can strongly influence patient outcomes, despite the use of the same IOL type. At present, no direct comparison of the axial and rotational stability of a (plate-haptic) toric IOLs after combined phacovitrectomy and sole phacoemulsification is available. Therefore, in the present study, we compare the refractive outcome, the accuracy of refractive prediction using different formulas and the axial and rotational stability of one specific plate-haptic toric IOL type (AT Torbi 709 M, Carl Zeiss Meditec AG, Jena, Germany) between combined phacovitrectomy and phacoemulsification alone.

## 2. Materials and Methods

### 2.1. Study Design

This retrospective study at the Department of Ophthalmology, University Hospital, LMU Munich, Munich, Germany, comprised 60 eyes of 60 patients who either underwent sole phacoemulsification with the implantation of a plate-haptic toric intraocular lens (n = 30) or combined phacovitrectomy with the implantation of a plate-haptic toric IOL (n = 30). The approval for the data collection and analysis was obtained from the local institutional review board of the Ludwig Maximilian University (approval number: 22-0591). This study complies with the principles outlined in the Declaration of Helsinki. The data collected were anonymized, removing any personal identifiable information.

Eyes with an age-related cataract and regular corneal astigmatism ≥1.0 diopters (D) which underwent small-incision phacoemulsification were included in the phacoemulsification-only group. Medical indications for combined phacovitrectomy were rhegmatogenous retinal detachment, an epiretinal membrane, a full-thickness macular hole, or a vitreous hemorrhage. Exclusion criteria included prior eye surgery, eye injuries, corneal pathologies and postoperative retinal complications. Only one eye per patient was included in the study. If both eyes met the study criteria, one eye was randomly selected. The regularity and magnitude of corneal astigmatism was preoperatively assessed by topographic Scheimpflug analysis (Pentacam^®^, Oculus Optikgeräte GmbH, Wetzlar, Germany). Optical biometry measurements and IOL power calculations were performed using the IOLMaster 700 (Carl Zeiss Meditec AG, Jena, Germany). Barret TK Toric, Haigis-T, or Z CALC formulas were used for IOL power calculations. The target refraction was calculated using all three formulas.

### 2.2. Surgery

Two experienced cataract and vitreoretinal surgeons (S.G.P. or T.C.K.) carried out all phacoemulsifications with toric intraocular lens implantation (AT Torbi 709 M, Carl Zeiss Meditec AG), followed by 25-gauge pars plana vitrectomy. The IOL utilized in this study is a bitoric, aspheric, one-piece IOL with an optic diameter of 6 mm, a total diameter of 11 mm and no haptic angulation. The material consists of hydrophilic acrylate with hydrophobic surface properties.

Cataract surgery was performed using a 2.5 mm clear corneal incision on the steep astigmatism axis and a curvilinear capsulorhexis between 4.5 and 5.5 mm. Following phacoemulsification, irrigation–aspiration was carried out for complete lens cortex removal. The IOL was inserted into the capsular bag using a single-use injector. After IOL insertion, the CALLISTO eye and Z ALIGN digital tracking system (Carl Zeiss Meditec AG) was used for intraoperative axis alignment. Incisions were sealed with stromal hydration using a balanced salt solution.

Pars plana vitrectomy was performed according to the underlying retinal disease, consisting of a core vitrectomy followed by vitreous removal in the periphery with globe indentation. For endotamponade, either air, gas (C2F6 or SF6 gas tamponade), or 5000 centistoke silicone oil was used. The sclerotomies were closed with absorbable sutures. Afterwards, axis alignment was repeated if necessary, using the digital tracking system as described above.

### 2.3. Outcome Measures

Ocular examination, including fundoscopy, spherical equivalent (SE) measurements, best corrected visual acuity (BCVA) assessment and optical biometry measurements were performed preoperatively and postoperatively (within 12 to 24 weeks after surgery). The postoperative IOL axis was determined by slit lamp examination. The straight positioning of the patient’s head was ensured by the examiner. The measuring eyepiece of the slit lamp with an integrated angular scale allowed for IOL axis determination with a resolution of 1°. The prediction error was defined as the postoperative SE minus the predicted SE calculated using Barrett TK Toric, Haigis-T and Z CALC 2.0 [16]. All of the formulas use Total Keratometry values directly measured by the IOL Master. The mathematics behind these formulas is incompletely available to the public; formulas may differ in incorporating factors, such as preoperative anterior chamber depth, lens thickness and cylinder power calculation methods (“net astigmatism”, “two-meridian approach”). ZCALC was specifically adapted to the IOLs designed by Zeiss. A positive PE corresponds to a more hyperopic result while a negative PE indicates a myopic shift. The anterior chamber depth (ACD) was set as the distance from the anterior corneal surface to the anterior natural lens/IOL surface, which was obtained by biometry. Postoperative ACD was also defined as the anatomical lens position (ALP) [17]. Surgically induced astigmatism (SIA) was calculated according to Alpin’s method [18].

### 2.4. Statistical Analysis

The data are presented as mean ± standard deviation. Statistical analysis was performed using Prism 8 (GraphPad Software Version 10.1.0, San Diego, CA, USA). A *p*-value < 0.05 was considered statistically significant. Intergroup differences were assessed using either an unpaired *t*-test or Mann–Whitney test according to a normality test. To assess the statistical significance of anterior chamber depth and axis misalignment between phacovitrectomy and phacoemulsification alone, both pre- and postoperatively, a two-way ANOVA followed by Šídák’s multiple comparisons test was performed.

## 3. Results

### 3.1. Patient Demographics

In total, 60 eyes of 60 patients were included. In the phacoemulsification-only group (n = 30), patients were significantly older (71.9 ± 7.8 years, *p* = 0.0008; Table 1) and showed a better visual acuity of 0.3 ± 0.3 logMAR compared to the phacovitrectomy group (n= 30; 0.5 ± 0.7 logMAR, *p* = 0.0002; Table 1). The indications for combined phacovitrectomy were an epiretinal membrane (n = 14), retinal detachment (n = 13), vitreous hemorrhage (n = 2) and a full-thickness macular hole (n = 1). Further baseline patient demographics and characteristics are depicted in Table 1.

### 3.2. Visual Acuity

In eyes with combined phacovitrectomy, preoperative BCVA improved significantly from 0.5 ± 0.7 logMAR to 0.2 ± 0.2 logMAR (*p* = 0.009) postoperatively. Similarly, in eyes with phacoemulsification alone, preoperative BCVA increased from 0.3 ± 0.2 logMAR to 0.1 ± 0.2 logMAR (*p* = 0.0002). Both preoperative and postoperative BCVA was significantly better in the eyes subjected to phacoemulsification alone (preoperative: mean difference 0.3 logMAR, *p* = 0.03; postoperative: mean difference 0.2 logMAR, *p* = 0.002).

### 3.3. Refractive Outcomes and Prediction Error

**Figure 1:**
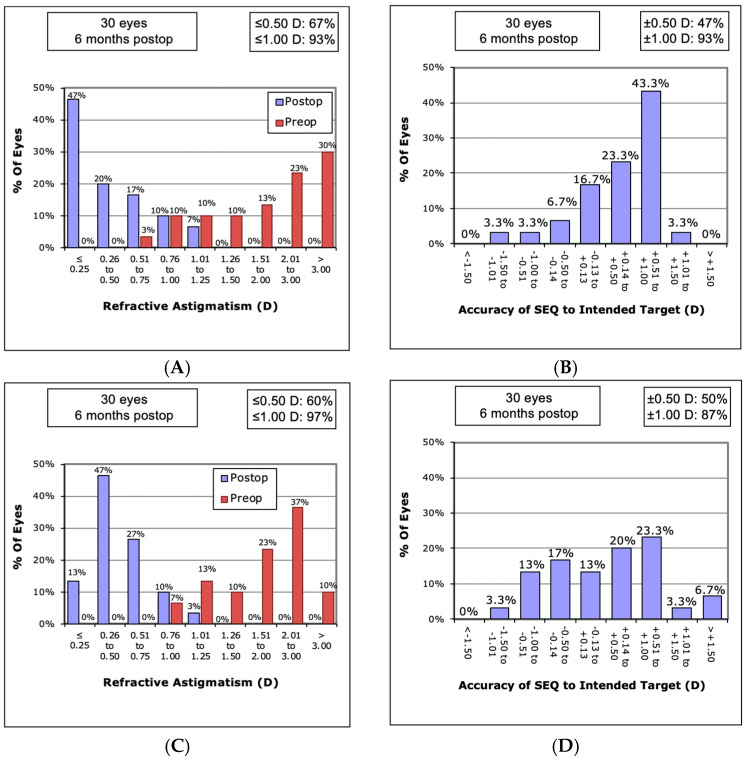
Refractive results after phacoemulsification alone and phacovitrectomy. (**A**) Amplitude of astigmatism before and after phacoemulsification. (**B**) Accuracy of spherical equivalent refraction before and after phacoemulsification. (**C**) Amplitude of astigmatism before and after phacovitrectomy. (**D**) Accuracy of spherical equivalent refraction before and after phacovitrectomy.

The mean spherical equivalent decreased considerably from −2 ± 4.4 D to −0.6 ± 1.4 D (target refraction either 0 or −2.5 D) after phacovitrectomy (*p* = 0.054) and −0.7 D ± 5.5 D to −0.1 ± 1.1 D after phacoemulsification (*p* = 0.54). Following phacovitrectomy and sole phacoemulsification, preoperative absolute astigmatism was significantly reduced from 2.9 ± 4.2 D to 0.6 ± 0.2 D (*p* = 0.004) and from 2.5 ± 1.4 D to 0.5 ± 0.3 D (*p* < 0.0001), respectively (Figure 1A,C). The overall mean prediction error was 0.34 ± 0.49 D after phacoemulsification and 0.23 ± 0.73 D after phacovitrectomy. The mean prediction errors (PE) for each formula after phacovitrectomy and phacoemulsification alone are depicted in Table 2. After phacovitrectomy, a mean surgically induced astigmatism (SIA) of 0.5 ± 0.4 D was observed, which was comparable to that after phacoemulsification (0.5 ± 0.2 D; *p* = 0.8)

### 3.4. Anatomical Lens Position and Axis Alignment

The anatomical lens position (ALP) after phacovitrectomy and phacoemulsification was 4.7 ± 0.3 mm and 4.5 ± 0.5, respectively (*p* = 0.07), with a comparable absolute ALP change (1.4 mm vs. 1.4 mm, *p* = 0.96). No significant difference was observed between both groups preoperatively (3.3 mm vs. 3.1 mm, *p* = 0.08). The postoperative ALP after phacovitrectomy with gas or air endotamponade did not differ (4.7 mm 4.6 mm, difference 0.1, *p* = 0.4). The mean axis misalignment after phacovitrectomy was 2.9 ± 2.9° postoperatively, whereas the mean axis misalignment after phacoemulsification was 2.1 ± 2° (mean difference 0.9° six months postoperatively, *p* = 0.5)

## 4. Discussion

This is the first study to compare the axial and rotational stability of plate-haptic toric IOLs after combined phacovitrectomy and sole phacoemulsification showing similar refractive result precision. Our data are consistent with recent studies showing no myopic shift in the postoperative refractive outcome after combined phacovitrectomy compared to phacoemulsification alone [10,19]. Phacovitrectomy was previously associated with a slight overcorrection of approximately −0.5 D, often related to inaccurate axial length measurements [3,6,20]. Notably, an axial length measurement error of 0.1 mm induces a postoperative refractive error of 0.27 D [21]. In our study, we used a state-of-the-art optical biometry device measuring the axial length from the anterior corneal surface to the retinal pigment epithelium; no significant difference in axial length measurements after phacovitrectomy compared to the baseline was found (mean absolute difference 0.04 D, *p* = 0.9).

The endotamponade pushing the IOL forward may also result in a myopic shift [9,22]. However, we observed no significant difference in the anatomical lens position (ALP) between the phacovitrectomy and phacoemulsification groups. Notably, approximately half of the eyes were subjected to air endotamponade, and its relatively fast resolution may have led to the consecutive backward movement of the IOL. In contrast, the anterior IOL displacement can be more permanent in eyes receiving gas endotamponade due to capsular fibrosis progression, as the gas takes three to six weeks to resolve after surgery [23]. However, we found no significant difference in ALP between the eyes receiving air compared to those with gas (SF6 and C2F6) endotamponade; however, our sample size is limited.

We compared three new-generation IOL calculation formulas commonly used for phacoemulsification with the implantation of a toric IOL. Overall, no significant difference in PE among these formulas was observed between the phacoemulsification and phacovitrectomy groups. Considering the PE as proxy for the anatomical lens position (ALP), all formulas predicted a slightly more anterior IOL position for both phacoemulsification and phacovitrectomy. We found that the prediction error (PE), while not statistically significant, leaned slightly more positively in the phacoemulsification than the phacovitrectomy group. The degree varied depending on the formula used for IOL power calculation. While this did not result in a significant myopic shift in our study, its consistency is noteworthy. This trend may be related to replacing the vitreous body with aqueous, which has a slightly lower refractive index (1.336 vs. 1.3346), contributing to a minor but consistent overcorrection in refraction, a factor unrelated to axial length measurement and postoperative IOL position. However, due to the limited size of the study, no definite conclusions can be drawn.

Generally, the different findings on a possible myopic shift after phacovitrectomy implicate multiple factors influencing postoperative refractive outcomes. Consequently, Falkner-Radler et al. linked different underlying posterior-segment pathologies to various degrees of postoperative myopic shift [9]. Similarly, preoperatively decreased visual acuity can lead to inaccurate axial length measurements due to poor fixation [9,10,19].

The implanted IOL type, including material, haptic design and angulation, may greatly affect postoperative intraocular stability. Thus, a sharp-edge design and haptic angulation were reported to induce axial IOL movement, causing deviations from the predicted target refraction [24], whereas a four-haptic angulated IOL was significantly less anteriorly displaced compared to a two-haptic non-angulated IOL after phacovitrectomy with gas endotamponade [4]. Moreover, Kim et al. found a single-piece non-angulated acrylic IOL to be inferior with respect to axial stability after phacovitrectomy compared to a three-piece IOL with a ten-degree haptic angulation [25]. The different studies illustrate the complexity when comparing different IOL types concerning axial and rotational stability. The results can also vary with the surgeon and eyes selected for analysis. Here, we chose an IOL for its (1) high flexibility due to the acrylic material facilitating IOL insertion, (2) hydrophobic surface properties that promote adherence and reduce the likelihood of early horizontal rotation and (3) four-point, balanced contact to the capsular bag that was previously shown to exert high intracapsular stability [26,27]. We previously showed that the rotational stability of the adopted plate-haptic IOL was comparable to previous studies using a loop-haptic IOL after phacovitrectomy and phacoemulsification alone [15]. Here, we further observed no significant difference in axis misalignment between the two groups, indicating that the procedure, be it phacovitrectomy or phacoemulsification, has no significant impact on the horizontal stability of our plate-haptic IOL.

We observed no significant difference in surgically induced astigmatism (SIA) after phacovitrectomy compared to sole phacoemulsification, which was similar to previous studies that reported SIA after phacovitrectomy of 0.23 ± 0.19 and 0.46 ± 0.37 [26,27]. No relevant differences to the phacoemulsification control group were found in either study [28,29]. The relatively posterior position of the scleral sutures likely minimizes the impact on the corneal curvature [28]. Consequently, the clear cornea incision (CCI) size was shown to have a greater impact on postoperative SIA after phacovitrectomy, consistent with sole phacoemulsification [30]. Thus, the absolute change in SIA of 0.5 D in our study was comparable to those that used a similar CCI size [30,31].

This study is limited due to its retrospective format. Different postoperative time points from two to six months were used for refractive results, axial position and optical biometry assessment, disregarding postoperative variations. Moreover, baseline differences within the phacovitrectomy and phacoemulsification groups can contribute to outcome variability. Additionally, the limited sample size complicates subgroup analysis. Only eyes were included for which postoperative biometry data were available, which may represent an additional confounder.

In conclusion, the prediction of the refractive outcome and intraocular lens position is similar for the plate-haptic toric AT Torbi 709M IOL after combined phacovitrectomy and phacoemulsification. Furthermore, the SIA was comparable between both groups. Accordingly, toric plate-haptic IOLs can confidently be used in phacovitrectomy without compromising refractive outcomes for the patient.

## Figures and Tables

**Table 1:** Baseline patient characteristics.

	Phacoemulsification	Phacovitrectomy	*p*-Value
Sex (female–male; n, %)	16:14 (53.3%)	11:19 (36.7%)	0.4
Mean age ± SD (years)	71.9 ± 7.8	64.8 ± 7.8	0.0008
Mean BCVA ± SD (logMAR)	0.3 ± 0.3	0.5 ± 0.7	0.0002
Spherical equivalent ± SD (D)	−0.43 ± 5.1	−2.0 ± 4.4	0.2
Axial length (mm) ± SD	24.1 ± 1.7	24.7 ± 1.6	0.1
Lens thickness (mm) ± SD	4.6 ± 0.4	4.5 ± 0.6	0.8
Anterior chamber depth (mm)	3.1 ± 0.4	3.3 ± 0.4	0.08
Total Keratometry (TK)1 (D)	41.9 ± 1.7	42 ± 1.3	0.8
Total Keratometry (TK)2 (D)	44.2 ± 2.1	43.8 ± 1.2	0.4
IOL power (SE)	19.2 ± 5.2	17.8 ± 4.7	0.3

Abbreviations: BCVA: best corrected visual acuity; IOL: intraocular lens.

**Table 2:** Prediction Error after phacovitrectomy and phacoemulsification.

Formula	Phacoemulsification		Phacovitrectomy		*p*-Value
	Predicted SE	Prediction Error (PE)	Predicted SE	Prediction Error (PE)	Difference in PE
Haigis-T	−0.32 ± 0.89	0.3 ± 0.78	−0.79 ± 1.05	0.19 ± 0.77	0.8
Barrett TK Toric	−0.19 ± 0.83	0.17 ± 0.84	−0.66 ± 0.97	0.06 ± 0.78	0.8
Z CALC	−0.5 ± 0.89	0.49 ± 0.73	−0.91 ± 1.07	0.32 ± 0.74	0.7

## Data Availability

The data presented in this study are available on request from the corresponding author due to privacy reasons.
